# Dopamine D_3_ Receptor Antagonism Reverses the Escalation of Oxycodone Self-administration and Decreases Withdrawal-Induced Hyperalgesia and Irritability-Like Behavior in Oxycodone-Dependent Heterogeneous Stock Rats

**DOI:** 10.3389/fnbeh.2019.00292

**Published:** 2020-01-14

**Authors:** Giordano de Guglielmo, Marsida Kallupi, Sharona Sedighim, Amy H. Newman, Olivier George

**Affiliations:** ^1^Department of Psychiatry, University of California, San Diego, La Jolla, CA, United States; ^2^Molecular Targets and Medications Discovery Branch, National Institute on Drug Abuse, Intramural Research Program, Baltimore, MD, United States

**Keywords:** VK4-116, escalation, opioid, dependance, withdrawal

## Abstract

Prescription opioids, such as oxycodone, are highly effective analgesics for clinical pain management, but approximately 25% of patients who are prescribed opioids misuse them, and 5%–10% develop an opioid use disorder (OUD). Effective therapies for the prevention and treatment of opioid abuse and addiction need to be developed. The present study evaluated the effects of the highly selective dopamine D_3_ receptor antagonist VK4-116 ([R]-*N*-[4-(4-[3-chloro-5-ethyl-2-methoxyphenyl]piperazin-1-yl)-3-hydroxybutyl]-1*H*-indole-2-carboxamide) on oxycodone addictive-like behaviors. We used a model of extended access to oxycodone self-administration and tested the effects of VK4-116 on the escalation of oxycodone self-administration and withdrawal-induced hyperalgesia and irritability-like behavior in male and female rats. Pretreatment with VK4-116 (5–25 mg/kg, i.p.) dose-dependently decreased the escalation of oxycodone self-administration and reduced withdrawal-induced hyperalgesia and irritability-like behavior in opioid-dependent rats. These findings demonstrate a key role for D_3_ receptors in both the motivation to take opioids and negative emotional states that are associated with opioid withdrawal and suggest that D_3_ receptor antagonism may be a viable therapeutic approach for the treatment of OUD.

## Introduction

More than 2 million Americans currently suffer from substance use disorders that are related to prescription opioid pain relievers, including oxycodone (Oxycontin^®^, Roxycodone^®^, Oxecta^®^), and 500,000 are addicted to heroin (Substance Abuse and Mental Health Services Administration, 2013). Over the past 20 years, the consumption of oxycodone increased by ~500%, and opioid-related overdose deaths quadrupled (Kolodny et al., 2015; Compton et al., 2016). Although opioid medications effectively treat acute pain and help relieve chronic pain for some patients (Moore et al., 2013), their use presents a dilemma for healthcare providers because of the risk of addiction. Only three treatments have been approved by the United States Food and Drug Administration for the treatment of opioid use disorder (OUD): methadone, buprenorphine, naltrexone, and their combination (e.g., Suboxone) or extended-release formulations (e.g., Vivitrol). Methadone is a synthetic opioid compound that binds primarily to the μ-opioid receptor (MOR), and buprenorphine is a MOR partial agonist. Methadone and buprenorphine are particularly effective in reducing opioid-induced mortality and maintaining patients in treatment, but important safety concerns and strict regulations because of their agonist properties at MORs have limited their use (National Academies of Sciences, Engineering, and Medicine, 2018). Naltrexone has lower efficacy and poor treatment adherence that have limited its real-world effectiveness (Ndegwa et al., 2016). The recent introduction of extended-release naltrexone (Vivitrol) has slightly improved the use of naltrexone compared with previous formulations (Ndegwa et al., 2016). New treatments for OUD with better efficacy and without agonistic effects at the MOR need to be developed.

Compelling evidence from animal studies suggests the involvement of dopamine D_3_ receptors in the development of addictive behaviors that are caused by many drugs of abuse, including opioids (Heidbreder and Newman, 2010; Sokoloff and Le Foll, 2017). D_3_ receptor antagonism has been shown to attenuate nicotine (Ross et al., 2007), oxycodone (You et al., 2019), and cocaine (Xi et al., 2005) self-administration, conditioned place preference (CPP) that is induced by drugs of abuse (Ashby et al., 2003; Song et al., 2013), and the reinstatement of drug-seeking behavior that is triggered by drug priming (Vorel et al., 2002; Andreoli et al., 2003; Xi and Gardner, 2007), stress (Xi and Gardner, 2007), and drug-associated cues (Aujla and Beninger, 2005; Higley et al., 2011; Galaj et al., 2015). Moreover, D_3_ receptor antagonism reduces locomotor sensitization and reverses the lowering of brain stimulation reward thresholds that is induced by drugs of abuse (Sonntag et al., 1992; Pak et al., 2006; Spiller et al., 2008; Higley et al., 2011). In contrast, D_3_ receptor antagonism does not affect the seeking of natural rewards, such as sucrose self-administration and sexual activity (Sonntag et al., 1992; Clément et al., 2009; Higley et al., 2011; You et al., 2017). However, all of these studies were conducted in animals that were subjected to limited-access opioid self-administration (e.g., 3 h/day). The efficacy of D_3_ receptor antagonism in animals that exhibit the escalation of opioid intake and opioid dependance remains to be determined. Therefore, the present study tested the effects of the highly selective D_3_ receptor antagonist VK4-116([R]-*N*-[4-(4-[3-chloro-5-ethyl-2-methoxyphenyl]piperazin-1-yl)-3-hydroxybutyl]-1*H*-indole-2-carboxamide; *in vitro* profile: D_3_ receptor *K*_i_ = 6.8 nM, 1,700-fold greater selectivity for D_3_ receptors vs. D_2_ receptors, and metabolic stability in mouse microsomes; Kumar et al., 2016; You et al., 2019) on oxycodone self-administration and withdrawal-induced hyperalgesia and irritability-like behavior in rats. The rats were given extended access to oxycodone self-administration, a model with high face validity, predictive validity, and construct validity for OUD (Zhang et al., 2014; Wade et al., 2015). This model is highly relevant to substance use disorders (Edwards and Koob, 2013; George et al., 2014) and associated with neuroadaptations that are also observed in humans with substance use disorders (Adinoff et al., 1990; Briand et al., 2008; George et al., 2008, 2012; Vendruscolo et al., 2012). The escalation model has been shown to exhibit seven of the 11 criteria of the *Diagnostic and Statistical Manual of Mental Disorders*, 5th edition (DSM-5; American Psychiatric Association, 2013), including most of the criteria that are required for severe use disorder: (1) tolerance (Ben-Shahar et al., 2006); (2) withdrawal (Ahmed et al., 2002; Vendruscolo et al., 2011); (3) substance taken in larger amount than intended (Ahmed and Koob, 1998); (4) unsuccessful efforts to quit (Ahmed and Cador, 2006; Lenoir et al., 2007); (5) considerable time spent to obtain the drug (Wee et al., 2008); (6) important social, work, or recreational activities given up because of use (George et al., 2008; Lenoir et al., 2013); and (7) continued use despite adverse consequences (Vanderschuren and Everitt, 2004; Vendruscolo et al., 2012; Xue et al., 2012; Seif et al., 2013). The use of extended-access oxycodone self-administration in animal strains that exhibit large individual differences, such as heterogeneous stock (HS) rats (Hansen and Spuhler, 1984; Woods and Mott, 2017), may provide new insights into the development of VK4-116 as a potential treatment for OUD.

The effect of VK4-116 on oxycodone intake (i.e., the primary endpoint in the present study to evaluate treatment efficacy) was tested in two independent cohorts of rats using different experimental designs (between-subjects and within-subjects) to confirm reproducibility. The effects of VK4-116 on hyperalgesia and irritability-like behavior that were associated with oxycodone withdrawal were tested using a within-subjects design.

## Materials and Methods

### Animals

Male and female HS rats were generated at the National Institutes of Health in the 1980s to encompass as much genetic diversity as possible by outbreeding eight inbred rat strains (ACI/N, BN/SsN, BUF/N, F344/N, M520/N, MR/N, WKY/N, and WN/N; Hansen and Spuhler, 1984). The HS rats (*n* = 60, *n* = 50 for the between-subjects experiment, *n* = 10 for the within-subjects experiment) were provided by Dr. Leah Solberg Woods (Medical College of Wisconsin, now at Wake Forest University School of Medicine) and housed two per cage on a reverse 12 h/12 h light/dark cycle (lights off at 8:00 AM) in a temperature (20–22°C) and humidity (45%–55%) controlled vivarium with *ad libitum* access to tap water and food pellets (PJ Noyes Company, Lancaster, NH, USA). All of the procedures were conducted in strict adherence to the National Institutes of Health *Guide for the Care and Use of Laboratory Animals* and were approved by the Institutional Animal Care and Use Committee of The Scripps Research Institute. At the time of testing, the rats’ body weights ranged between 350 and 400 g.

### Intravenous Catheterization

The animals were anesthetized by isoflurane inhalation, and intravenous catheters were aseptically inserted in the right jugular vein using a modified version of a procedure that was described previously (de Guglielmo et al., 2015, 2017). The vein was punctured with a 22-gauge needle, and the tubing was inserted and secured inside the vein by tying the vein with suture thread. The catheter assembly consisted of an 18 cm length of Micro-Renathane tubing (0.023-inch inner diameter, 0.037-inch outer diameter; Braintree Scientific, Braintree, MA, USA) that was attached to a guide cannula (Plastics One, Roanoke, VA, USA). The guide cannula was bent at a near right angle, embedded in dental acrylic, and anchored with mesh (2 cm square). The catheter exited through a small incision on the back, and the base was sealed with a small plastic cap and metal cover cap. This design helped to keep the catheter base sterile and protected. The catheters were flushed daily with heparinized saline (10 U/ml of heparin sodium; American Pharmaceutical Partners, Schaumburg, IL, USA) in 0.9% bacteriostatic sodium chloride (Hospira, Lake Forest, IL, USA) that contained 20 mg/0.2 ml of the antibiotic Timetin (GlaxoSmithKline, Brentford, UK).

### Operant Training

Self-administration was performed in operant conditioning chambers (29 cm × 24 cm × 19.5 cm; Med Associates, St. Albans, VT, USA) that were enclosed in sound-attenuating, ventilated environmental cubicles. The front door and back wall of the chambers were constructed of transparent plastic, and the other walls were opaque metal. Each chamber was equipped with two retractable levers that were located on the front panel. Oxycodone was delivered through plastic catheter tubing that was connected to an infusion pump. The infusion pump and a cue light were activated by responses on the right (active) lever. Responses on the left (inactive) lever were recorded but did not have any scheduled consequences. Activation of the pump resulted in the delivery of 0.1 ml of the fluid. A computer controlled fluid delivery and behavioral data recording.

### Oxycodone Self-administration

Each session was initiated by the extension of two retractable levers into the operant chamber. Responses on the right active lever were reinforced on a fixed-ratio 1 (FR1) schedule by intravenous oxycodone (150 μg/0.1 ml/kg/infusion) that was infused over 6 s, followed by a 20 s timeout period that was signaled by the illumination of a cue light above the active lever. Daily 12-h sessions were conducted for 15 days (five sessions/week). Responses on the left inactive lever were recorded but had no scheduled consequences. The animals had access to food but not water during the 12-h session.

### Drugs

Oxycodone (Sigma Aldrich, St. Louis, MO, USA) was dissolved in 0.9% sodium chloride (Hospira, Lake Forest, IL, USA) and administered at a dose of 150 μg/0.1 ml/kg. The dose of oxycodone was selected based on previous studies (Wade et al., 2015; Nguyen et al., 2019) and because it produces significant plasma oxycodone concentrations (40 ng/ml; Mavrikaki et al., 2017). VK4-116 was synthesized by Kumar et al. (2016) in the Newman laboratory at the National Institute on Drug Abuse, based on a published procedure. VK4-116 was dissolved in 25% 2-hydroxypropyl-β-cyclodextrin and intraperitoneally injected at doses of 0, 5, 15, and 25 mg/kg as previously reported (You et al., 2019).

### Mechanical Nociceptive Von Frey Testing

Mechanical nociception, reflected by hind paw withdrawal thresholds, was determined by an observer who was blind to the experimental conditions using von Frey filaments, ranging from 8.511 to 281.838 g. The test was performed similarly to previous studies (Kallupi et al., 2018; Kononoff et al., 2018). The test began after 10 min of habituation to the testing environment. A series of von Frey filaments were applied from below the wire mesh to the central region of the plantar surface of the left hind paw in ascending order of force, beginning with the smallest filament (8.511 g). The filament was applied until buckling of the hair occurred, and the filament remained in place for 2 s. Rapid withdrawal of the hind paw was considered a positive response. The stimulus was incrementally increased until a positive response was observed and then decreased until a negative response was observed to determine a pattern of responses to apply to previously described statistical methods (Dixon, 1980). Once the threshold was determined for the left hind paw, the same testing procedure was applied to the right hind paw after 5 min. The 50% paw withdrawal threshold was determined by the formula *Xf + kδ*, where *Xf* is the last von Frey filament applied, *k* is the Dixon value that corresponded to the response pattern, and *δ* is the mean difference between stimuli. Paw withdrawal thresholds were determined for rats before self-administration (baseline) and 12 h after the last self-administration session (12-h withdrawal).

### Irritability-Like Behavior

To test irritability-like behavior during oxycodone withdrawal, we used the bottle-brush test, based on the methods of Lagerspetz and Portin (1968) and Riittinen et al. (1986) and modified slightly for rats (Kimbrough et al., 2017). Irritability-like behavior was tested 12 h after the last self-administration session (12-h withdrawal). Irritability-like behavior was examined by measuring aggressive and defensive responses during the bottle-brush test. Irritability-like behavior sessions were conducted in a randomized order for each animal. Testing consisted of 10 trials per rat in plastic cages (10.5 inches × 19 inches × 8 inches; Ancare, Bellmore, NY, USA) with fresh bedding. During each trial, the rat started at the back of the cage. A bottle brush was rotated toward the animal’s whiskers (from the front of the cage) by a treatment-naive experimenter. The brush was rotated around the whiskers of the rat for approximately 1 s. The brush was then rotated back to the front of the cage where it was allowed to hang vertically for approximately 2 s, during which behavioral responses were recorded. A 10-s intertrial interval was used. Three observers who were blind to treatment scored the behaviors in real-time. For each rat, separate sums of aggressive and defensive responses across all trials were determined for each observer. Aggressive and defensive response scores for each rat were then calculated by averaging the observers’ sums. This was then used to calculate a group mean and SEM. The following were scored as aggressive responses: smelling the target, biting the target (during the initial phase of rotating the brush forward and back to the starting position), boxing the target, following the target, exploring the target (using paws or mouth to manipulate the brush without biting or boxing), mounting the target, and delayed biting (during the 2 s that the brush hung at the starting position). The following were scored as defensive responses: escaping from the target, digging, burying, defecation, jumping, climbing, vocalization, and grooming. Grooming and digging were additionally recorded during the 10-s intertrial intervals.

### Effect of VK4-116 on the Escalation of Oxycodone Self-administration (Between-Subjects)

Rats (*n* = 50, 25 males and 25 females) were trained to self-administer oxycodone under an FR1 schedule of reinforcement in daily 12-h sessions. Each active lever press resulted in the delivery of one oxycodone infusion (150 μg/kg/0.1 ml infusion). A 20-s timeout period followed each oxycodone infusion. During the timeout period, responses on the active lever did not have scheduled consequences. This timeout period occurred concurrently with the illumination of a cue light that was located above the active lever to signal delivery of the positive reinforcement. The rats were trained to self-administer oxycodone in fourteen 12-h sessions (5 days/week). At this point, the rats were divided into four groups (*n* = 12/13 group with similar intake) and intraperitoneally injected with VK4-116 (0, 5, 15, and 25 mg/kg) 30 min before beginning the session.

### Effect of VK4-116 on the Escalation of Oxycodone Self-administration and Withdrawal-Induced Hyperalgesia and Irritability-Like Behavior (Within-Subjects)

A separate group of rats (*n* = 10, four males and six females) were trained to self-administer oxycodone as described above. After 14 sessions of oxycodone self-administration, the animals were intraperitoneally injected with VK4-116 (0, 15, and 25 mg/kg) in a counterbalanced order to evaluate the effects of VK4-116 on withdrawal-induced hyperalgesia using the von Frey test. At the end of the hyperalgesia test, the animals were allowed to self-administer oxycodone so that the effects of VK4-116 on self-administration were assessed again using a Latin-square design. The animals were subjected to oxycodone self-administration at 2-day intervals between drug tests. At the end of the Latin square, the rats were baselined again for oxycodone self-administration and intraperitoneally injected with VK4-116 (0 and 25 mg/kg) in a counterbalanced order to evaluate the effects of VK4-116 on withdrawal-induced irritability-like behavior.

### Statistical Analysis

The self-administration data were analyzed using repeated-measures analysis of variance (ANOVA) of the number of infusions that were earned during the escalation interval. The effects of VK4-116 on self-administration and hyperalgesia were analyzed using appropriate one- or two-way ANOVAs (between- or within-subjects) according to the experimental design. The irritability-like behavior data were analyzed using Student’s *t*-test. Significant effects in the ANOVA were followed by the Newman–Keuls *post hoc* test. Values of *p* < 0.05 were considered statistically significant.

## Results

### Effect of VK4-116 on the Escalation of Oxycodone Self-administration

#### Between-Subjects Study

**Figure 1:**
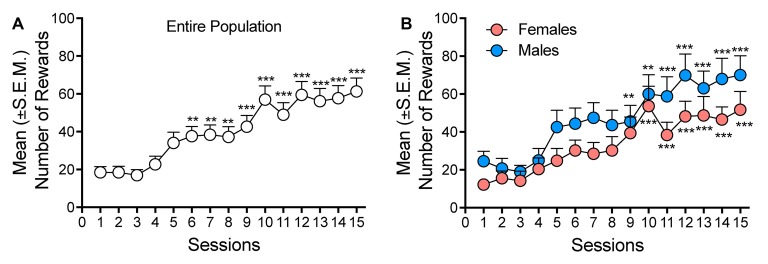
**(A)** Escalation of oxycodone intake in heterogeneous stock (HS) rats. **p < 0.01, ***p < 0.001, vs. day 1. **(B)** Escalation of oxycodone intake in male (blue) and female (salmon) HS rats. **p < 0.01, ***p < 0.001, vs. day 1.

**Figure 2:**
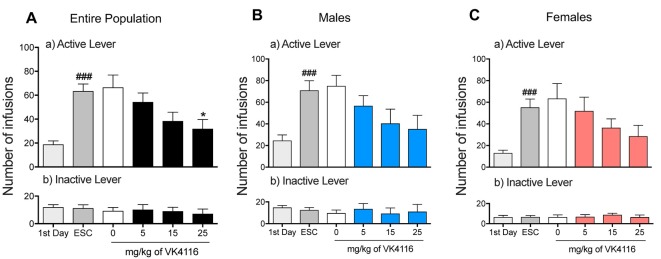
**(A)** Effect of VK4116 on the escalation of oxycodone self-administration in HS rats. **(B)** Effect of VK4116 on the escalation of oxycodone self-administration in male HS rats. **(C)** Effect of VK4116 on the escalation of oxycodone self-administration in female HS rats. The upper panels (a) represent responses at the active lever. The lower panels (b) represent responses at the inactive lever. *p < 0.05, vs. 0 mg/kg; ^###^*p* < 0.001, vs. first day of oxycodone self-administration.

After 3 weeks of oxycodone self-administration, the rats gradually escalated their oxycodone intake (one-way ANOVA, *F*_(14,658)_ = 15.46, *p* < 0.001). The Newman–Keuls *post hoc* test revealed the significant escalation of oxycodone self-administration that began from session 6 until session 15 compared with the first day of extended access (*p* < 0.01 for sessions 6–8; *p* < 0.001 for sessions 10–15; Figure 1A). The two-way ANOVA that also incorporated sex as a variable indicated that both males and females escalated their oxycodone intake, with a significant main effect of time (*F*_(14,644)_ = 15.15, *p* < 0.001). However, no differences in oxycodone intake were observed between males and females, reflected by the lack of a main effect of sex (*F*_(1,46)_ = 2.738, *p* > 0.05) and no sex × time interaction (*F*_(1,46)_ = 0.614, *p* > 0.05; Figure 1B). Treatment with VK4-116 significantly decreased operant responding for oxycodone (*F*_(3,44)_ = 3.454, *p* < 0.05). The Newman–Keuls *post hoc* test revealed that VK4-116 reduced oxycodone self-administration at the dose of 25 mg/kg (*p* < 0.05, 25 mg/kg vs. 0 mg/kg; Figure 2A). The two-way ANOVA that also incorporated sex as a variable indicated that VK4-116 significantly reduced oxycodone intake in males and females, reflected by a significant main effect of treatment (*F*_(3,39)_ = 4.203, *p* < 0.05), with no main effect of sex (*F*_(1,39)_ = 0.703, *p* > 0.05) and no sex × treatment interaction (*F*_(3,39)_ = 0.004, *p* > 0.05; Figures 2B,C). Inactive lever responding was low and unaltered by VK4-116 treatment (Figures 2A–C, bottom panels).

**Figure 3:**
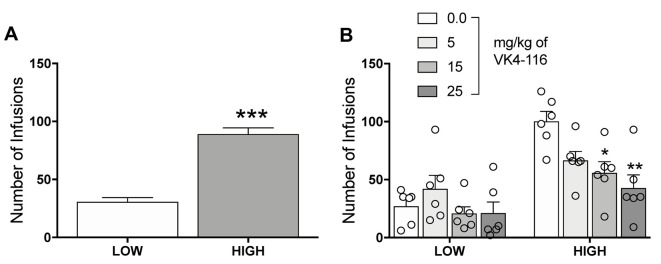
**(A)** Oxycodone self-administration in HS rats with high vs. low intake. ***p < 0.001. **(B)** Effect of VK4116 in HS rats with high vs. low oxycodone intake. *p < 0.05, **p < 0.01, vs. 0 mg/kg.

Further analysis of the data allowed us to identify two different subpopulations of rats. Using a median split, we were able to divide the rats into High and Low responders based on their level of oxycodone self-administration (unpaired *t*-test, *t*_46_ = 8.897, *p* < 0.001; Figure 3A). The data on the effects of VK4-116 on oxycodone self-administration were then re-analyzed based on the effects of VK4-116 on these two subpopulations, the results of which showed that VK4-116 reduced oxycodone intake selectively in High-responder rats. The two-way ANOVA, with group (High and Low) and treatment (0, 5, 15, and 25 mg/kg) as between-subjects factors, showed significant effects of group (*F*_(1,40)_ = 36.3, *p* < 0.001) and treatment (*F*_(3,40)_ = 5.143, *p* < 0.01) and a significant group × treatment interaction (*F*_(3,40)_ = 3.441, *p* < 0.05). The Newman–Keuls *post hoc* test revealed that VK4-116 reduced oxycodone self-administration at doses of 15 mg/kg (*p* < 0.05) and 25 mg/kg (*p* < 0.01) selectively in High-responder rats (Figure 3B).

#### Within-Subjects Study

**Figure 4:**
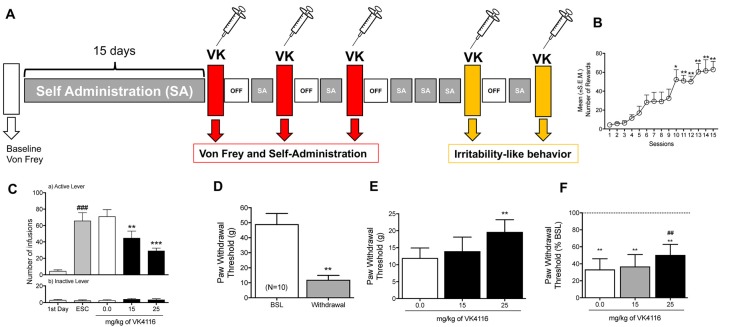
**(A)** Schematic diagram of the experiment. **(B)** Escalation of oxycodone intake in HS rats. *p < 0.05, **p < 0.01, vs. day 1. **(C)** Effect of VK4116 in HS rats (within-subjects design). The upper panel (a) represents responses at the active lever. The lower panel (b) represents responses at the inactive lever. **p < 0.01, ***p < 0.001, vs. 0 mg/kg; ^###^*p* < 0.001, vs. first day of oxycodone self-administration. **(D)** Development of mechanical hyperalgesia after the escalation of oxycodone self-administration in HS rats. **p < 0.01, vs. baseline (BSL). **(E,F)** Effect of VK4116 on oxycodone withdrawal-induced hyperalgesia. The data are presented as grams of force applied. **(E)** **p < 0.01, vs. 0 mg/kg and as a percent change from baseline pre-oxycodone. **(F)** **p < 0.01, vs. BSL; ^##^*p* < 0.01, vs. 0 mg/kg.

After 3 weeks of oxycodone self-administration, the rats gradually escalated their oxycodone intake (one-way ANOVA, *F*_(14,126)_ = 14.25, *p* < 0.0001). The Newman–Keuls *post hoc* test revealed the significant escalation of oxycodone self-administration that began from session 10 until session 15 compared with the first day of extended access (*p* < 0.05 for session 10; *p* < 0.001 for sessions 11–15; Figures 4A,B). The two-way ANOVA that also incorporated sex as a variable indicated that both males and females escalated their oxycodone intake, reflected by a significant main effect of time (*F*_(14,112)_ = 12.53, *p* < 0.001, data not shown), with no main effect of sex (*F*_(1, *8*)_ = 0.3883, *p* > 0.05) and no sex × time interaction (*F*_(14,112)_ = 0.3832, *p* > 0.05, data not shown). These results confirmed the observations in the between-subjects experiment that treatment with VK4-116 significantly decreased operant responding for oxycodone (*F*_(2,18)_ = 13.68, *p* < 0.001). The Newman–Keuls *post hoc* test revealed that VK4-116 reduced oxycodone self-administration at both doses tested (*p* < 0.001, 25 mg/kg vs. 0 mg/kg; *p* < 0.0001, 25 mg/kg vs. 0 mg/kg; Figure 4C). Inactive lever responding was low and unaltered by VK4-116 treatment (Figure 4C, bottom panel).

### Effect of VK4-116 on the Negative Emotional State That Is Induced by Oxycodone Withdrawal

At 12 h of withdrawal after 15 sessions of extended-access oxycodone self-administration, the rats exhibited the development of hyperalgesia, reflected by lower paw withdrawal thresholds in the von Frey test compared with baseline pre-oxycodone thresholds (*t*_(9)_ = 4.825, *p* < 0.01; Figure 4D). The one-way ANOVA indicated that treatment with VK4-116 significantly reduced withdrawal-induced hyperalgesia (*F*_(2,18)_ = 7.017, *p* < 0.01). The Newman–Keuls *post hoc* test revealed that VK4-116 reduced withdrawal-induced hyperalgesia at the dose of 25 mg/kg (*p* < 0.01, 25 mg/kg vs. 0 mg/kg; Figure 4E). Figure 4F shows the data plotted as a percentage of baseline before oxycodone exposure, indicating that VK4-116 reduced withdrawal-induced hyperalgesia but not to levels of the baseline response.

**Figure 5:**
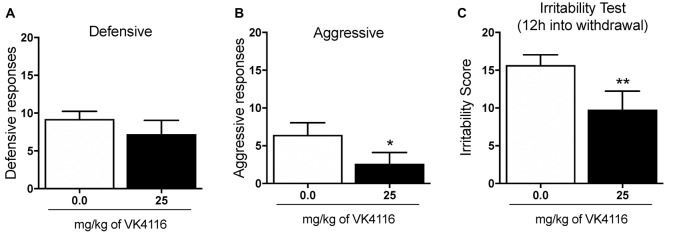
Effect of VK4116 on oxycodone withdrawal-induced irritability-like behavior. **(A)** Number of defensive responses. **(B)** Number of aggressive responses. **(C)** Total irritability score (defensive + aggressive). *p < 0.05, **p < 0.01, vs. 0 mg/kg.

At the end of this experiment, the animals were baselined again to determine their level of oxycodone self-administration and tested for the effects of VK4-116 (25 mg/kg) on irritability-like behavior 12 h into withdrawal. Treatment with VK4-116 did not alter defensive responses (*t*_(9)_ = 1.212, *p* < 0.01; Figure 5A) but significantly reduced aggressive responses (*t*_(9)_ = 2.759, *p* < 0.01; Figure 5B) and the total irritability score (*t*_(9)_ = 3.484, *p* < 0.01; Figure 5C).

## Discussion

The present study used two different experimental designs (between- and within-subjects) and found that D_3_ receptor antagonism with VK4-116 dose-dependently reduced oxycodone self-administration in oxycodone-dependent male and female rats with a history of escalated oxycodone self-administration. The reduction of oxycodone intake was mainly driven by the effect of VK4-116 in a subpopulation of rats that exhibited a high intake phenotype (i.e., High responders). We then found that the effect of VK4-116 was not limited to drug intake and also extended to a reduction of withdrawal-induced hyperalgesia and irritability-like behavior in oxycodone-dependent rats.

The effects of VK-116 were similar in male and female rats and replicated using two different experimental designs (between- and within-subjects). VK4-116 partially reversed the escalation of oxycodone self-administration, which is consistent with previous studies that reported that D_3_ receptor antagonism decreased opioid self-administration in rats that were given limited access to oxycodone or heroin self-administration (Boateng et al., 2015; You et al., 2017; Jordan et al., 2019a) and decreased opioid-induced CPP in rats (Ashby et al., 2003). The present results replicated these findings and further demonstrated that VK4-116 was also effective in an animal model of opioid dependance.

The escalation of intake in opioid-dependent animals has been hypothesized to be driven by the development of a negative emotional state during opioid withdrawal (Koob, 2019). Opioid withdrawal is characterized by hyperalgesia, irritability-like behavior, and anxiety-like behavior (Edwards et al., 2012; Koob, 2019). We hypothesized that the effects that were observed in self-administration studies might be mediated by the ability of VK4-116 to alleviate the negative emotional state during opioid withdrawal. As expected, pretreatment with VK4-116 attenuated oxycodone withdrawal-induced hyperalgesia and irritability-like behavior. These results are consistent with previous studies that reported that VK4-116 dose-dependently attenuated naloxone-precipitated conditioned place aversion in chronic oxycodone-treated rats (You et al., 2019) and that the D_3_ receptor antagonist SB-277011A blocked naloxone-precipitated conditioned place aversion in chronic morphine-treated rats (Rice et al., 2012).

The inhibitory effects of VK4-116 on the escalation of oxycodone self-administration and withdrawal-induced hyperalgesia and irritability-like behavior are unlikely to be attributable to nonspecific motor impairment because a previous study that tested VK4-116 reported that it did not affect locomotor activity (Kumar et al., 2016; Jordan et al., 2019b). Moreover, treatment with VK4-116 and other D_3_ receptor antagonists did not alter operant sucrose self-administration (Vorel et al., 2002; Clément et al., 2009; Higley et al., 2011; You et al., 2017).

In summary, the present study found that VK4-116 decreased opioid self-administration and attenuated aversive states that were induced by oxycodone withdrawal. The negative emotional states that arise from drug withdrawal are considered to be major factors that drive compulsive drug taking and seeking in drug-dependent individuals (Koob and Mason, 2016; Koob and Volkow, 2016). VK4-116 reduced the reinstatement of drug-seeking behavior that was triggered by oxycodone priming (Jordan et al., 2019a). Despite evidence of the involvement of D_3_ receptors in drug addiction, the clinical translation of these findings has been challenging because of insufficient absorption, distribution, metabolism, and excretion properties of D_3_ antagonists and possible cardiotoxicity when they are administered in the presence of cocaine (Appel et al., 2015; Keck et al., 2015). The only highly selective D_3_ receptor antagonist that has been tested in humans is GSK598809, which was evaluated for the treatment of obesity, nicotine dependence, and alcohol dependence (Dodds et al., 2012; Mugnaini et al., 2013; Murphy et al., 2017). Acute GSK598809 administration produced a significant short-term reduction of nicotine craving (Mugnaini et al., 2013), providing the first clinical evidence that D_3_ receptor antagonism may be effective for the treatment of substance use disorders.

VK4-116 has been shown to be highly selective and have a stable metabolic profile across species (Kumar et al., 2016; Jordan et al., 2019a). This metabolic stability distinguishes VK4-116 from many previously characterized D_3_ receptor ligands and suggests better translational potential (Jordan et al., 2019a). Altogether, the present results and previous findings suggest that VK4-116 may be a promising pharmacotherapeutic agent for the treatment of OUD given its ability to reduce the motivation to take opioids and attenuate opioid withdrawal symptoms.

## Data Availability Statement

The datasets generated for this study are available on request to the corresponding author.

## Ethics Statement

The animal study was reviewed and approved by IACUC University of California San Diego.

## Author Contributions

GG designed and performed the research and wrote the manuscript. MK and SS performed the research. AN provided the compound. OG designed the research and wrote the manuscript.

## Conflict of Interest

The authors declare that the research was conducted in the absence of any commercial or financial relationships that could be construed as a potential conflict of interest.
